# Testing for variation in photoperiodic plasticity in a butterfly: Inconsistent effects of circadian genes between geographic scales

**DOI:** 10.1002/ece3.11713

**Published:** 2024-07-07

**Authors:** Olle Lindestad, Sören Nylin, Christopher W. Wheat, Karl Gotthard

**Affiliations:** ^1^ Department of Ecology, Environment and Plant Sciences Stockholm University Stockholm Sweden; ^2^ Department of Zoology Stockholm University Stockholm Sweden; ^3^ Bolin Centre for Climate Research Stockholm Sweden

**Keywords:** butterfly, candidate genes, circadian genes, diapause, insect, photoperiodism

## Abstract

The genetic components of the circadian clock have been implicated as involved in photoperiodic regulation of winter diapause across various insect groups, thereby contributing to adaptation to adverse seasonal conditions. So far, the effects of within‐population variation in these genes have not been well explored. Here, we present an experimental test of the effects of within‐population variation at two circadian genes, *timeless* and *period*, on photoperiodic responses in the butterfly *Pararge aegeria*. While nonsynonymous candidate SNPs in both of these genes have previously shown to be associated with diapause induction on a between‐population level, in the present experiment no such effect was found on a within‐population level. In trying to reconcile these results, we examine sequence data, revealing considerable, previously unknown protein‐level variation at both *timeless* and *period* across Scandinavian populations, including variants unique to the population studied here. Hence, we hypothesize that these variants may counteract the previously observed diapause‐averting effect of the candidate SNPs, possibly explaining the difference in results between the experiments. Whatever the cause, these results highlight how the effects of candidate SNPs may sometimes vary across genetic backgrounds, which complicates evolutionary interpretations of geographic patterns of genetic variation.

## Graphical abstract

Circadian clock genes have been observed to correlate with the seasonal control of insect development and dormancy across a number of insect species. Here we follow up on previous results in the speckled wood butterfly, where population differences in seasonal development depended on variation in two candidate genes. We fail to replicate this effect at a within‐population level, suggesting that the effect may be modulated by local genetic background.

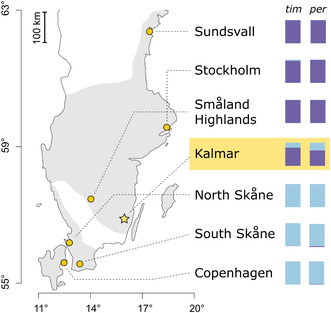

## 1 INTRODUCTION

Photoperiod, the relative length of day versus night, changes predictably across the year at a given latitude. Consequently, many plants and animals utilize photoperiod as a temporal cue, signaling the appropriate time for mating, molting, migration, or other life cycle events with a high degree of year‐to‐year accuracy (Bradshaw & Holzapfel, 2007). In insects, perhaps the most important photoperiodic trait is diapause, a hormonally programmed state of suppressed development and heightened stress tolerance that functions as an adaptation to adverse seasonal conditions, e.g. during winter (Koštál, 2006; Tauber et al., 1986). Although details vary, a wide range of insects and other arthropods use photoperiod (e.g. short days in the fall) as a cue to control the entry into diapause. In some species, photoperiod also regulates exit from diapause, or pre‐ and post‐diapause development rates (Beck, 1980; Gotthard et al., 2000). Because both climate and daylength patterns vary with geographic location, photoperiodic traits tend to be subject to local adaptation: populations experience divergent selection for daylength reaction norms that produce locally optimal life cycles (Hut et al., 2013).

Genetic variation in life cycle regulation in turn affects how populations respond, both on ecological and evolutionary time scales, to changes in cues or conditions, e.g. during range shifts or as a result of climate change (Forrest & Miller‐Rushing, 2010; Visser et al., 2010). This has inspired several decades of study into the genetics of photoperiodic plasticity, and especially the regulation of diapause. A notably common result, across a wide range of insect species and investigative methods (Goto, 2013; Hodková et al., 2003; Iiams et al., 2019; Pavelka et al., 2003; Sakamoto et al., 2009; Saunders, 1990), has been the apparent involvement of circadian genes in the regulation of development in general, and diapause in particular. Circadian genes and their protein products form the molecular components of the circadian clock, which in insects includes two sets of core oscillator genes that reciprocally regulate one another in a 24‐h negative feedback loop. The core positive oscillators are *clock* and *cycle*, while the core negative oscillators are *timeless, period*, and usually *cryptochrome2* (Meuti & Denlinger, 2013; Yuan et al., 2007). Results connecting diapause induction to circadian genes are especially interesting in light of the long‐standing debate over whether the insect circadian clock itself, as a functional unit, is involved in photoperiodism, or whether photoperiodism and circadian function are separate systems that merely share genes in common (Bradshaw & Holzapfel, 2010; Koštál, 2011; Meuti & Denlinger, 2013; Saunders et al., 2004).

Allelic variation in circadian genes has been tied to geographic variation in diapause traits between wild insect populations. The core circadian genes *timeless* and *period* in particular have frequently emerged as candidates: variation in *timeless* has been connected to geographic variation in diapause induction in two different fruit fly species (Tauber et al., 2007; Yamada & Yamamoto, 2011) as well as in pitcher plant mosquitoes (Mathias et al., 2007), while variation in *period* has been connected to geographic variation in diapause induction in a parasitic wasp (Paolucci et al., 2016) and a butterfly (Pruisscher et al., 2020), as well as to diapause termination in a moth (Kozak et al., 2019). Results from these systems point toward a key role for circadian genes in local adaptation across climate clines; however, the consequences for life cycle regulation of within‐population variation at circadian loci remain mostly unexplored.

Traits such as overall diapause rate, diapause duration, and photoperiod thresholds for inducing or terminating diapause, are quantitative and have shown the potential to evolve quickly under new selective regimes (Bradshaw & Holzapfel, 2001; Ittonen et al., 2022; Nielsen et al., 2023). This includes several cases of adaptation from small founder populations (Bean et al., 2012; Lehmann et al., 2015; Urbanski et al., 2012; Yamanaka et al., 2008), suggesting considerable standing within‐population variation in diapause regulation. Still, this does not preclude the presence of a few variable loci which explain a notably large share of the geographic variation in diapause traits (Fu et al., 2015; Lehmann et al., 2016; Mathias et al., 2007; Pruisscher et al., 2017). Identifying genetic loci with effects on diapause traits may allow for recent selective regimes or future adaptive potential to be inferred from patterns of allele frequencies across different populations. This is a potentially powerful approach; see Levy et al. (2015) for a very successful example. However, drawing general conclusions from allele frequencies at a few loci across a geographic cline rests on the assumption that a given genetic variant has the same effect across different genetic backgrounds, which may not be the case.

An opportunity to test this assumption is presented by the speckled wood butterfly, *Pararge aegeria*. In this species, Pruisscher et al. (2018) uncovered a small set of loci, including *timeless* and *period*, that showed strong between‐population differentiation and low within‐population variation, indicating divergent selective sweeps across a north‐south climate cline in Scandinavia. This discovery prompted a phenotypic test of second‐generation hybrid offspring between a northern and a southern population, which showed that rates of diapause induction were correlated with the nonsynonymous SNP variation at *timeless*, and possibly *period* (Pruisscher et al., 2018) Hence, there is good reason to believe that variation at these two loci, especially *timeless*, is involved in generating the variation in diapause regulation seen across these populations (early diapause in the north; late diapause in the south). Here we build on these results by investigating a *P. aegeria* population located at intermediate latitudes, which harbors allelic variation at both *timeless* and *period*. Our aim is to test whether the same circadian gene candidate SNPs that appear to drive diapause variation on a regional scale (Pruisscher et al., 2018) also affect diapause induction on a more homogeneous genetic background within a single population—and, if such an effect is not found, to investigate potential reasons why.

## 2 MATERIALS AND METHODS

### 2.1 Study species

*Pararge aegeria* is a woodland‐associated satyrine butterfly with a complex but well‐characterized life cycle (Lindestad et al., 2021; Nylin et al., 1989, 1995). Photoperiod affects development in several ways, of which two are of interest here. Firstly, pupal diapause is induced in individuals that experience short days during the end of the larval stage (Friberg et al., 2011). Secondly, larval development is polyphenic, being molded by photoperiod into two main modes, “fast” and “slow” (Lindestad et al., 2021). “Fast” development is induced by long days, and takes less than a month. “Slow” development is induced by short days, varies continuously from 40 to 80 days depending on photoperiod, and is much less sensitive to variation in temperature (Nylin et al., 1989). “Fast” larvae tend to develop into nondiapausing pupae. Nonetheless, the two decisions are partly independent, at least under static laboratory daylengths: the switch between larval modes occurs at longer daylengths than the switch between diapause and nondiapause (Lindestad et al., 2019; Nylin et al., 1989, 1995). Furthermore, the larval development response appears to have shifted in response to recent climate change, while the pupal diapause response has not (Nielsen et al., 2023). These two photoperiodic responses, acting in concert over the season, help *P. aegeria* populations express a life cycle suitable to the local climate (Lindestad et al., 2019).

**FIGURE 1:**
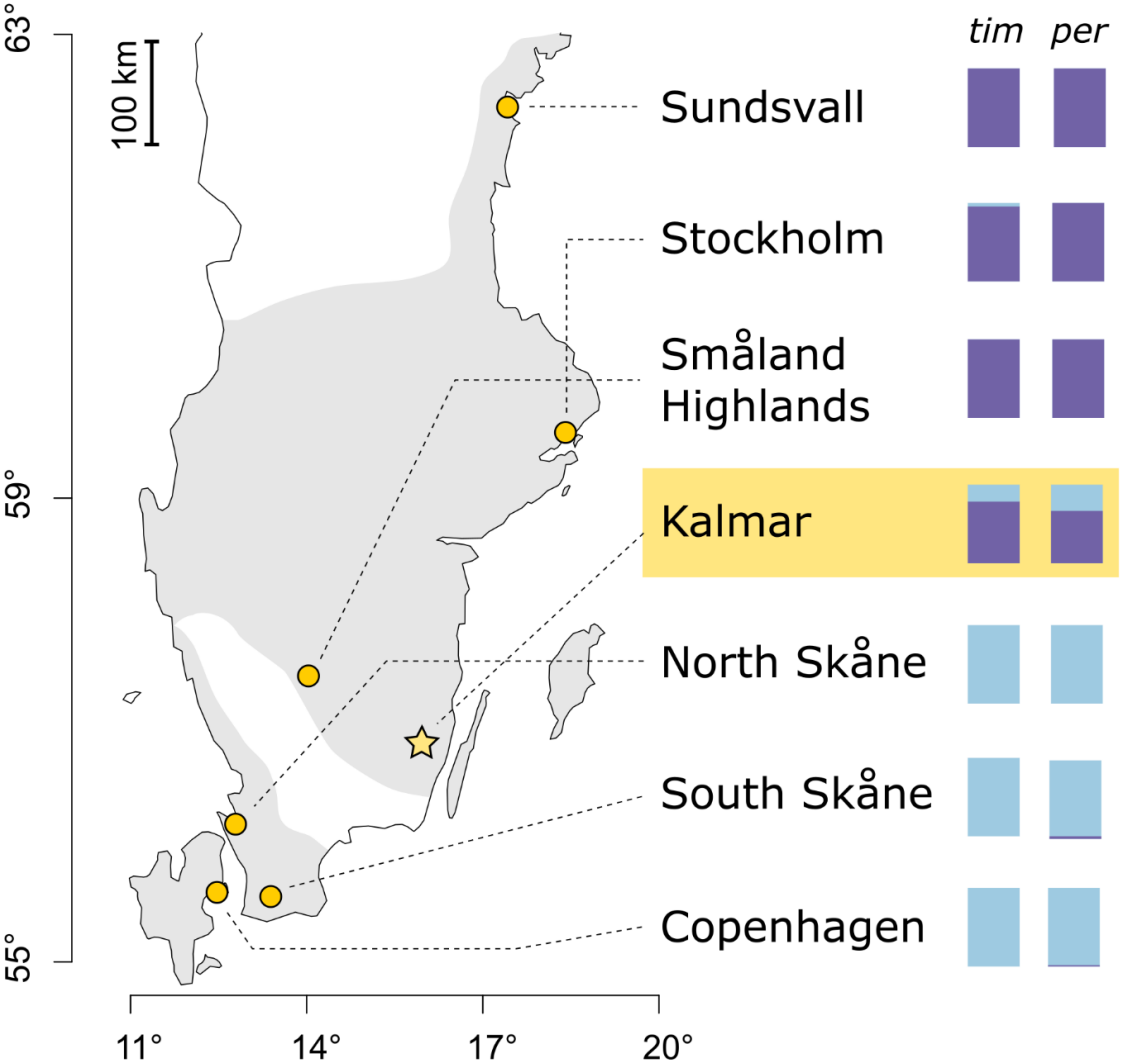
Partial map of Sweden and Denmark, showing *P. aegeria* distribution (shaded area). The sampling location for the photoperiod experiment is marked with a star. Bars show local allele frequencies at the candidate SNPs in *timeless* (*tim*) and *period* (*per*), which were previously shown to correlate with photoperiodic plasticity in a cross between *P. aegeria* from Sundsvall and North Skåne (Pruisscher et al., 2018). The variant in *period* spans two adjacent nucleotides, but these segregate together, hence only one frequency value is shown.

Swedish mainland populations of *P. aegeria* are split, based on genetic background and colonization history, into a northern and a southern cluster (Lindestad et al., 2022; Tison et al., 2014). These clusters are separated by a range gap within which *P. aegeria* is rare or absent (Eliasson et al., 2005; Nordström, 1955) (Figure 1). The northern mainland populations produce one generation per year, while the southern populations produce two; this is enabled by divergent photoperiodic life cycle adaptations (Aalberg Haugen & Gotthard, 2015; Lindestad et al., 2019; Nylin et al., 1995).

### 2.2 Candidate SNPs

As shown by Pruisscher et al. (2018), northern and southern populations of *P. aegeria* in Sweden are nearly fixed for alternate alleles of a nonsynonymous SNP in *timeless*, and the same is true for two immediately adjacent SNPs (altering two adjacent amino acids) in *period*. These SNPs (Figure 1) will hereafter be termed the “candidate SNPs,” partly to distinguish them from additional SNPs in these two genes that will be discussed later in this text. In a phenotypic test using a cross between two populations at opposite ends of the Swedish range, the “southern” (*S*) alleles at these candidate SNPs were associated with higher rates of “fast” larval development (and hence, by extension, lower rates of diapause induction). This was especially true for the SNP in *timeless* (Pruisscher et al., 2018).

The subject of the present study is a population located in Kalmar county, at the southern edge of the northern cluster's range. Kalmar is notable in that the candidate SNPs in *timeless* and *period* are variable: Both the “southern” (S) and “northern” (N) alleles are present at intermediate frequencies (Figure 1). Despite this, at the whole‐genome level, the Kalmar population shows no greater similarity to the southern population cluster than do any populations further north, indicating little or no recent gene flow across the distribution gap (Lindestad et al., 2022). Presumably, this means that the N and S alleles of *timeless* and *period* have been segregating in the population for many generations, allowing for a test of the phenotypic effect of the candidate SNPs on an otherwise similar genetic background.

### 2.3 Butterfly sampling and genotyping

On May 30th, 2017, five adult female *P. aegeria* were collected from a forest in Kalmar county (56.93°N; 16.04°E). A thin strip of tissue (~0.5 cm^2^) was cut from each hindwing, and DNA was extracted from these tissue samples using the standard “cell and tissue DNA” kit on a KingFisher Duo Prime purifier (ThermoFisher Scientific, Waltham, MA, USA). Genotypes at the candidate SNPs in *timeless* and *period* were determined using High Resolution Melt (HRM). The primer pairs used (one per gene) were designed for an earlier study (Pruisscher et al., 2018). For each primer pair, the forward and reverse primer were located 25 bp upstream and downstream of the target SNP. The HRM reaction was carried out in 96‐well reaction plates, using a reaction mix consisting of 5 μL MeltDoctor HRM Master Mix (ThermoFisher Scientific, Waltham, MA, USA), 3.3 μL milliQ H_2_O, 0.6 μL of each primer pair, and 1 μL sample (2–7 ng DNA/μL). Individuals with known genotypes (heterozygote as well as both homozygotes), in duplicate, were used as controls. Both the amplification and melt steps were performed on a StepOnePlus Real‐Time PCR system (Applied Biosystems, Waltham, MA, USA). The amplification step consisted of heat activation at 95°C for 10 min, followed by 40 cycles of elongation at 95°C for 15 s and annealing at 60°C for 1 min. For the melt step, temperature was increased from 60 to 95°C at 1% ramp speed, and fluorescence was measured every 0.1°C. Melt curves were analyzed using High Resolution Melt Software version 3.0.1 (Applied Biosystems). Individuals were assigned a genotype at each locus by visual comparison to the melt curves of the control individuals. Samples that yielded ambiguous melt curves were re‐analyzed once; if results were still unclear, this individual was not used.

As is common for lepidopterans, *period* in *P. aegeria* is located on the Z chromosome, meaning females have only one copy (Pruisscher et al., 2018). However, since heterozygotes were not used in this study, and homo‐ and hemizygous states are assumed to be functionally interchangeable, little distinction will be made in this text. For the sake of simplicity, when referring to allelic state at *period* we use “homo/hemizygote” broadly to refer to any individual possessing only one of the two alleles (p^N^ or p^S^), in either one copy (females) or two (males).

### 2.4 Rearing and crosses

All five wild‐caught females had mated in the field with unknown males, and laid eggs in the laboratory. Egg‐laying took place in individual cages on tufts of bluegrass (*Poa* sp.). Approximately 60 offspring per female were reared to adulthood, forming five F1 families (K1–K5). F1 larvae were reared in family cages on orchard grass (*Dactylis glomerata*), under a 20 L photoperiod (20 h light: 4 h dark), with temperatures of 16°C in the day phase and 14°C in the night phase. Emerging F1 adults were tissue sampled through wing clipping and genotyped as described above.

The main experiment was designed to test the effect of *timeless* genotype on a homogenous *period* background. F2 crosses were arranged so as to maximize sampling across families while restricting *timeless* genotype to one of the two homozygous states (t^N^t^N^ or t^S^t^S^) and limiting *period* variation to p^N^p^N^ (p^N^0 in females). Heterozygotes at either locus were not used. Because two F1 families contained t^N^t^N^ individuals, two contained t^S^t^S^ individuals, and the fifth family contained homo/hemizygotes of both alleles (Table 1), three between‐family crosses were possible for generating each type of *timeless* homozygote. Reciprocal crosses were conducted separately to control for sex‐linked effects, yielding twelve possible *timeless* crosses (Table 2). Each cross was carried out in a separate mating cage, using 1–3 individuals of each sex to ensure that mating occurred. It was not logistically feasible to observe each mating as it occurred, and multiple females may have contributed to the eggs laid within a cage. Hence, while individual parent identity is not known for each F2 individual, the F1 family that each parent belonged to is known and controlled for in the analyses.

**TABLE 1:** Genotypes present in the five F1 families, each derived from a wild‐caught mated female.

Family	Timeless	Period
K1	t^N^t^N^; t^N^t^S^; (t^S^t^S^)	p^N^p^N^; p^S^p^S^; p^N^p^S^
K2	t^S^t^S^; t^N^t^S^	p^N^p^N^
K3	t^S^t^S^; t^N^t^S^	p^N^p^N^
K4	t^N^t^N^; t^N^t^S^; t^S^t^S^	p^N^p^N^
K5	t^N^t^N^; t^N^t^S^	p^N^p^N^; p^S^p^S^; p^N^p^S^

*Note*: Period is sex‐linked and females are hemizygous for this locus; p^N^p^N^ or p^S^p^S^ in a female actually means a single copy of the respective allele (p^N^0 or p^S^0), but this is omitted in order to improve readability for this table. Family K1 contained only a few t^S^t^S^ individuals, probably because a limited number of the mother's eggs had been fertilized by a second male (none of those offspring were used in crosses).

**TABLE 2:** Setup of F2 crosses: numbers of F1 males/females in the mating cage, their family backgrounds, and offspring used.

Genotype	Mat. family	Pat. family	Females	Males	F2 larvae used
“Southern” *tim* (t^S^t^S^; p^N^p^N^/p^N^0)	K2	K3	3	2	36
	K2	K4	3	3	36
	K3	K2	2	2	36
	K3	K4	3	2	36
	K4	K2	2	2	36
	K4	K3	2	2	36
“Northern” *tim* (t^N^t^N^; p^N^p^N^/p^N^0)	K1	K4	1	2	36
	K1	K5	–	–	–
	K4	K1	2	1	36
	K4	K5	2	2	36
	K5	K4	2	2	36
	K5	K1	2	2	72
“Southern” *per* (t^N^t^N^; p^S^p^S^/p^S^0)	K5	K1	3	3	72

*Note*: The K1 × K5 cross was not carried out, due to a lack of appropriate breeding individuals. The main experiment (effect of *timeless*) is a comparison between all t^S^t^S^ p^N^p^N^ individuals and all t^N^t^N^ p^N^p^N^ individuals; the sub‐experiment (effect of *period*) is a comparison between the offspring of the two K5 × K1 crosses (bottom two rows). Note that only the relevant homo‐/hemizygotes from each family were used (see “genotype” column); heterozygotes at either locus were not used.

Of the twelve main crosses, one (K1 × K5) was not carried out: Because the K1 and K5 families were variable at *period*, there was an insufficient number of individuals with combinations of sex and genotype appropriate for the main experiment. However, the variation in these two families also allowed for an additional, smaller test of the effect of *period* genotype on a homogenous *timeless* background (t^N^t^N^). To this end, a cross was carried out using K5 females and K1 males. Results for this “sub‐experiment” cross (t^N^t^N^/p^S^p^S^) were compared to the K5 × K1 (t^N^t^N^/p^N^p^N^) individuals in the main experiment; sample size was doubled for both of these crosses to better enable the response to be estimated (Table 2).

### 2.5 Photoperiodic response phenotyping

Eggs were laid on bluegrass in the mating cages, and stored at 17°C under a 12 L photoperiod (12 h light; 12 h dark) until hatching. Within a day of hatching, F2 larvae were randomly assigned to one of three static photoperiod treatments: 17, 17.5 L or 18 L (all at 18°C). The photoperiod treatments were chosen to maximize variation in diapause decision. Specifically, treatments were roughly centered on the critical daylength of the Kalmar population, i.e. the daylength under which 50% of individuals enter diapause at pupation.

Larvae were reared in 1‐L plastic jars containing a living tuft of bluegrass in liquid nutrient solution. Each jar contained three larvae from the same cross, each cross was represented by two jars per climate cabinet, and each photoperiod treatment was represented by two climate cabinets, for a total of 12 larvae per cross and treatment. For the two K1 × K5 crosses there were instead four jars per cabinet, for a total of 24 larvae per cross and treatment (Table 2). Hence the initial sample size in the main experiment was 72 individuals per *timeless* genotype and treatment, and in the secondary experiment, 24 individuals per *period* genotype and treatment.

Fresh grass tufts were provided at regular intervals to ensure ad libitum food access, and jars were rotated within climate cabinets every 3 days to reduce location effects. Pupated individuals were transferred into individual jars to await adult eclosion. At 18°C, nondiapausing *P. aegeria* pupae typically develop to adulthood within 13 ± 2 days (Lindestad et al., 2020; Nylin et al., 1989), so a pupa that had failed to develop into an adult within 20 days was scored as having entered diapause. The development rate decision of young larvae (“fast” or “slow” mode) was scored depending on whether larval development rate was higher than 0.029 day^−1^ (i.e. whether pupation had occurred within 35 days). At threshold daylengths where both modes occur, this value corresponds to a sharp local minimum in the bimodal frequency distribution of development rates (cf. Nylin et al., 1989). Out of the 504 experimental individuals, 351 survived to pupation, and 323 pupae survived for the 20 days needed to score diapause status. Survival rates may have been reduced somewhat by competition, as larvae were reared in groups of three (which was done to improve sample size); however, mortality was similar across treatments and genotypes.

Twice during the experiment, a climate cabinet malfunction occurred where the lights did not switch off at night as programmed. The first malfunction was in one of the two 17.5 L climate cabinets, lasted for a week, and caused all larvae in this cabinet to rapidly develop into non‐diapausing pupae. All data from this cabinet were excluded from the pupal diapause analysis. The second malfunction was in one of the two 17 L cabinets, lasted only 4 days, and appears not to have affected the results: Only seven un‐pupated individuals were left in the cabinet at this point, and when they eventually pupated, they all entered diapause. An alternate statistical analysis excluding these seven individuals gave virtually indistinguishable results from those reported here. Fortunately, both cabinet malfunctions occurred several months after all fast‐developing larvae had pupated, hence the fast/slow response had already been recorded for all individuals. Therefore, all data from all cabinets could be used for the analysis of larval development mode. After the first major malfunction, cabinet function was monitored regularly; therefore, we deem it unlikely that any more malfunctions of this type affected the results of the experiment, especially given the similar responses across genotypes (see Section 3).

The final average sample size per treatment and genotype was, in the main experiment, 34.8 for pupal diapause, 50.5 for larval development mode; and in the sub‐experiment, 13.2 for pupal diapause, 17.8 for larval development mode. Full details are shown in Table 3.

**TABLE 3:** Final sample sizes (accounting for mortality) per genotype and photoperiod treatment in the photoperiodic assay.

Experiment	Trait analyzed	Genotype	17 L	17.5 L	18 L
Main experiment (effect of *tim*)	Diapause/nondiapause	t^N^t^N^	48	15	53
		t^S^t^S^	37	10	46
	Larval development mode	t^N^t^N^	53	51	56
		t^S^t^S^	47	47	49
Sub‐experiment (effect of *per*)	Diapause/nondiapause	p^N^p^N^/p^N^0	18	5	21
		p^S^p^S^/p^S^0	16	3	16
	Larval development mode	p^N^p^N^/p^N^0	19	18	22
		p^S^p^S^/p^S^0	18	13	17

*Note*: Sample sizes for 17.5 h of light were smaller when analyzing diapause/nondiapause, because of a climate cabinet failure that specifically affected the results for this trait.

**FIGURE 2:**
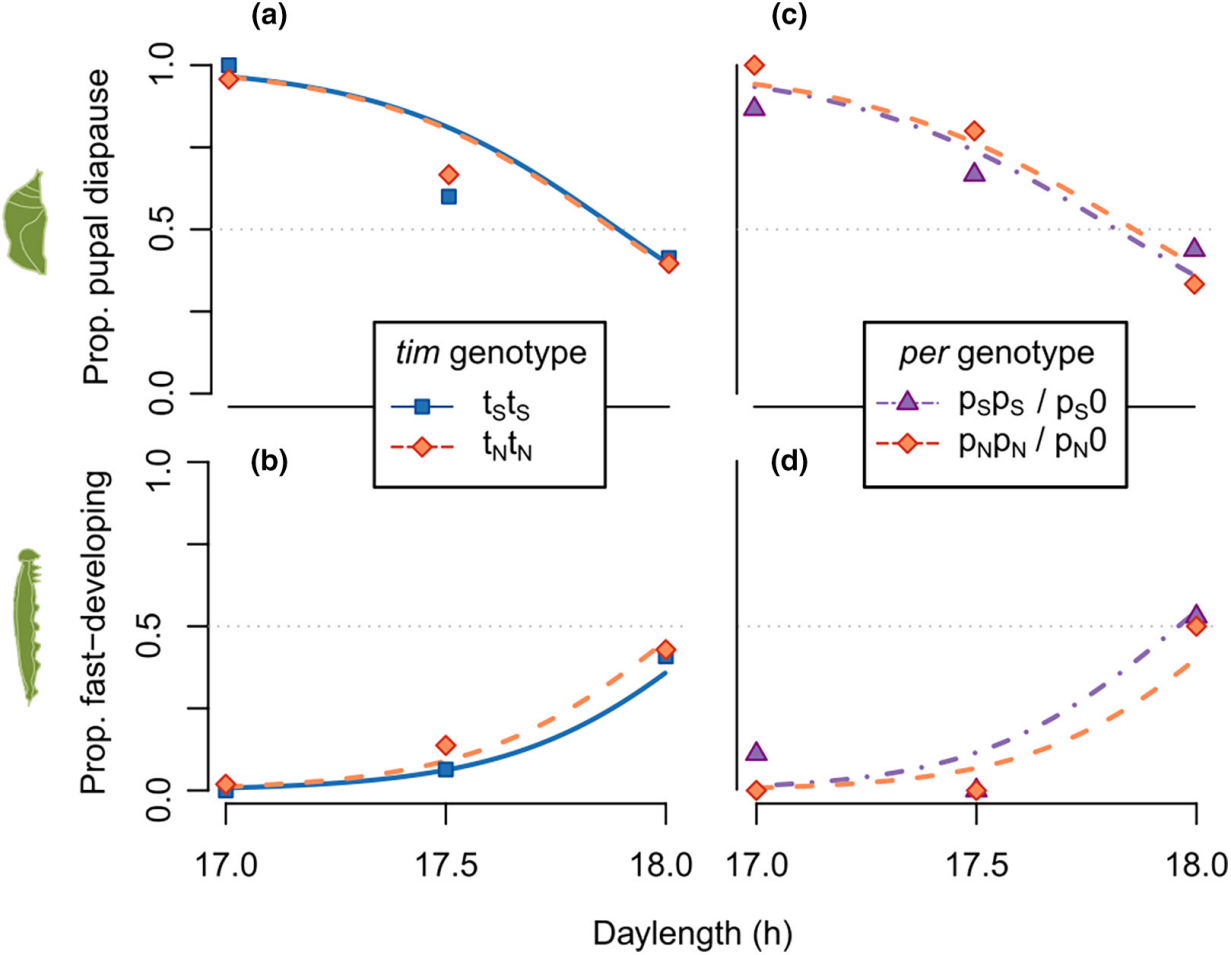
Effects of daylength and genotype on diapause incidence (top) and larval development mode (bottom). Points are proportions per treatment and genotype; lines are fitted probabilities from the GLMM analyses. (a, b) main experiment, effect of *timeless* on homogenous *period* background—blue squares and solid line for SS homozygotes; orange diamonds and dashed line for NN homozygotes. (c, d) sub‐experiment, effect of *period* on homogenous *timeless* background—purple triangles and dot‐dashed line for SS homozygotes; orange diamonds and dashed line for N homo/hemizygotes.

For the main experiment (effect of *timeless*), diapause decision and larval development mode were analyzed as binary traits using mixed‐effects generalized linear models (GLMMs). For both response traits, the fixed effects were daylength (scaled to mean = 0 and sd = 1), genotype at *timeless*, and the *time* × daylength interaction. Rearing jar, mother's family and father's family were used as random effects. The sub‐experiment (effect of *period*) was analyzed in a similar way, with *period* genotype, daylength and their interaction as main effects, and rearing jar as a random effect. (Because the sub‐experiment only used K1 × K5 crosses, all individuals' mothers and fathers came from the same families respectively.) The genotype × daylength interaction was found nonsignificant in all cases (*p* > .1), and analyses were re‐run with the interaction removed to avoid needlessly sacrificing statistical power (Engqvist, 2005). When testing larval development as a function of *period* genotype, the model failed to converge when the interaction was included, as a result of complete separation at some family levels. However, visual inspection of the results (Figure 2) strongly suggested a lack of interaction, i.e., the shape of the logistic response curve was similar for both genotypes.

### 2.6 Additional variation at *timeless* and *period*

To help interpret the results of the photoperiodic assay, sequence data for *timeless* and *period* were examined to check for the presence of additional amino acid variants. This was done using a whole‐genome dataset for the focal Kalmar population as well as six other Scandinavian populations: three to the north of Kalmar (the Småland Highlands, 57.5°N; Stockholm, 59.6°N; Sundsvall, 62.4°N), and three to the south (North Skåne, 56.3°N; South Skåne, 55.6°N; Copenhagen, 55.6°N). The dataset had been generated from DNA samples pooled from multiple individuals within each population, with the sequence data mapped to a de novo genome for *P. aegeria* (Lindestad et al., 2022). The Kalmar sample from the whole‐genome dataset (*n* = 13) was a separate sample from the founder females used for the rearing experiment. For a simple overview of amino acid variation, SNPs were called on a pileup file of all seven populations using *PoPoolation2* v1.201 (Kofler et al., 2011), with a minimum allele count of 5 and a minimum PHRED quality of 20, and all detected exonic SPNs in *timeless* and *period* were manually characterized as synonymous or nonsynonymous using the Integrative Genomics Viewer (Robinson et al., 2011). In the absence of detailed functional characterization of the protein domains of TIM and PER in butterflies, we used Grantham's distance to provide some sense of the likelihood that each given substitution has a phenotypic effect. This index, which takes a value between 5 and 215, is a coarse measure of the physicochemical dissimilarity between alternative amino acids (Grantham, 1974).

## 3 RESULTS

### 3.1 Photoperiodic response phenotyping

In the main experiment, genotype at the candidate SNP in *timeless* did not affect photoperiodic thresholds, with no effect on diapause incidence (analysis of deviance; ${\chi^{2}}_{1}$ = 0.026; *p* = .87), nor on larval development mode (analysis of deviance; ${\chi^{2}}_{1}$ = 0.73; *p* = .39). Likewise, the sub‐experiment showed no effect of *period* genotype on diapause incidence (analysis of deviance; ${\chi^{2}}_{1}$ = 0.044; *p* = .83), nor on larval development mode (analysis of deviance; ${\chi^{2}}_{1}$ = 0.67; *p* = .41). In fact, across all treatments, all genotypes were highly similar in terms of diapause and larval development proportions (Figure 2).

Diapause induction rates across treatments suggested a critical daylength for diapause induction of approximately 17.9 h. This estimate is similar to what has previously been observed for the Kalmar population, and is consistent with a univoltine life cycle (one generation per year) in the field (Lindestad et al., 2019).

### 3.2 Amino acid variation at *timeless* and *period*

**FIGURE 3:**
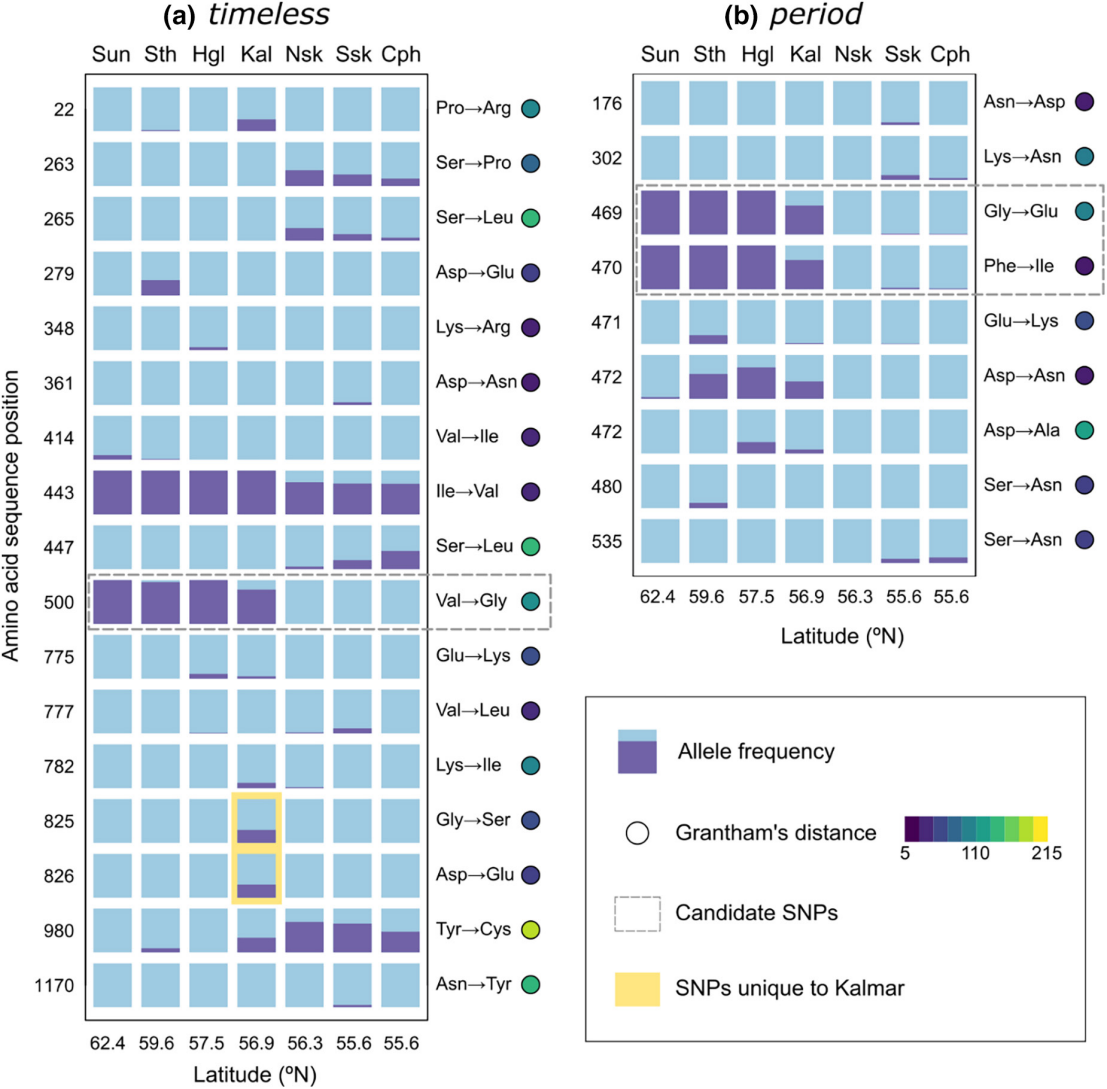
Bar plots showing allele frequencies of nonsynonymous SNPs in *timeless* (a) and *period* (b) for Kalmar (middle column) and six other populations: Sun = Sundsvall, Sth = Stockholm, Hgl = Småland Highlands, Nsk = North Skåne, Ssk = South Skåne, Cph = Copenhagen. Sun and Nsk were the populations used in the previous experiment by Pruisscher et al. (2018). Circles show Grantham's distance for each substitution; brighter color indicates a larger dissimilarity between the two alternative amino acids. The candidate SNPs tested in the experiment are marked with dashed rectangles; novel variants unique to Kalmar are marked with a yellow rectangle. In *period*, two SNPs altered the same codon, resulting in three alleles at position 472.

**FIGURE 4:**
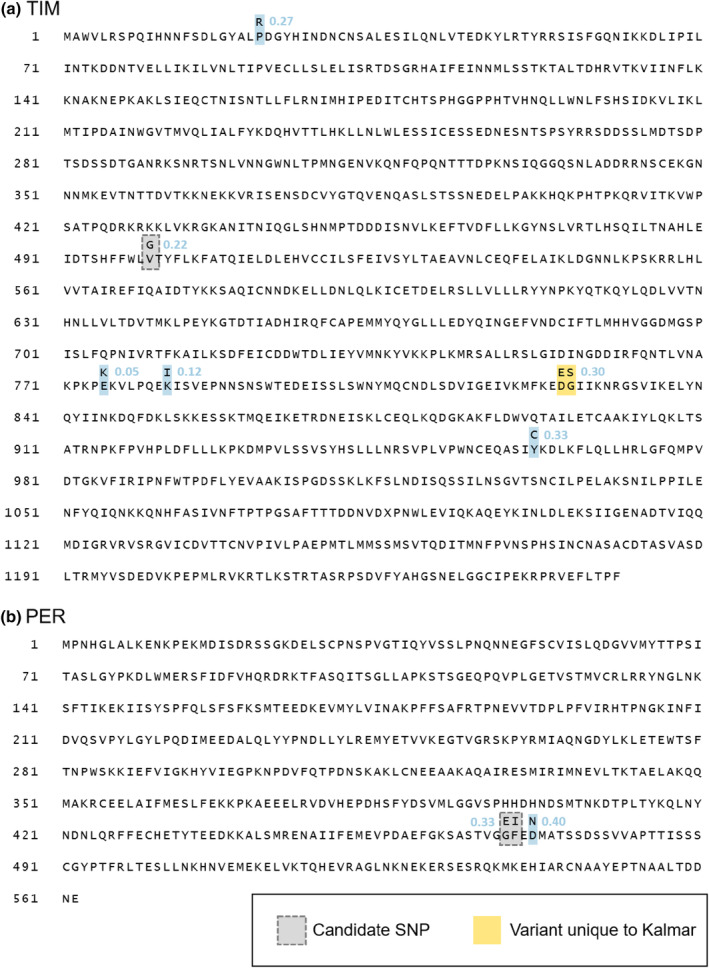
Predicted protein sequence for (a) *timeless* and (b) *period*, showing all amino acid polymorphisms found in the Kalmar population at a minimum allele count of five. Numbers show frequencies of the minor allele. The candidate variants first characterized by Pruisscher et al. (2018) are outlined in dashed gray; variants found only in Kalmar are highlighted in yellow. Note that only approximately half of the *period* sequence is shown (and was analyzed), as the rest of the *period* gene was split across multiple contigs in the genome assembly used.

To help interpret the results of the rearing experiment, we analyzed sequence data for *timeless* and *period*. This analysis revealed that, in addition to the variation at the candidate SNPs in these genes, there were large amounts of previously uncharacterized SNP variation in both genes, within the Kalmar population as well as across Scandinavian populations. For *timeless*, eight amino acid sites were polymorphic in Kalmar (Figure 3a). These included a linked substitution of two adjacent amino acids (Gly‐Asp to Ser‐Glu) found only in this population; the remaining variants were shared with one or several other populations. Novel variation was also found at *period*, with a common amino acid substitution just downstream of the candidate SNP (Figure 3b). Full amino acid sequences for both proteins, showing the location of Kalmar substitutions, are provided in Figure 4.

Curiously, for both genes, nearly all detected SNPs across the surveyed populations were nonsynonymous: 17 out of 19 for *timeless* and nine out of 10 for *period*. The frequencies of these SNPs varied between populations independently of one another, hence a wide range of relatively common variants of the TIM and PER proteins evidently exist across Scandinavian *P. aegeria*. The precise number of protein‐level alleles cannot be deduced, as pooled‐sequence data does not provide linkage information across the length of an entire gene. Although some of the detected substitutions were for highly similar amino acids (i.e. low value of Grantham's distance), several substitutions had intermediate or high values of Grantham's distance, suggesting at least a possibility of an effect on protein function (Figure 3).

## 4 DISCUSSION

Based on the highly similar responses of all genotypes in the photoperiodic assay (Figure 2), it seems that the amino acid variants at *timeless* and *period* characterized by Pruisscher et al. (2018) do not generate variation in diapause induction or larval development rate between individuals of the Kalmar population. On the genetic background tested here, both candidate SNPs appear to be phenotypically neutral. How can this result can be reconciled with the significant effect detected, especially for *timeless*, in Pruisscher et al.'s original cross experiment? There are at least two options (besides chance, or experimental error in either study). The first possibility is that the effect detected in the original study was not generated by variation in *timeless* or *period* as such, but rather by other variation in genetic linkage with these loci, which a laboratory cross would be unable to disentangle. This possibility cannot be ruled out based on our results. However, the fact that *timeless* and *period* have been implicated in diapause regulation across such distantly related insects as moths (Levy et al., 2015), flies (Tauber et al., 2007), and wasps (Dalla Benetta et al., 2019) speaks against such an association in *P. aegeria* being a coincidence.

The second possibility is that the candidate SNPs do not generate any variation in photoperiodic response between Kalmar individuals because of some additional, population‐specific variation (in *timeless*, in *period*, or at other loci) that negates or compensates for the effect of the candidate SNPs. Consistent with this explanation, follow‐up analyses of sequence data revealed that the Kalmar *P. aegeria* population does indeed contain additional, common nonsynonymous variants in both *timeless* and *period*, some of which are seemingly unique to this population (Figure 3). This complication was unexpected, as the novel variation was missing from both populations (Sun and Nsk) surveyed by Pruisscher et al. (2018), and the HRM analyses used to select genotypes for the present study only targeted the candidate SNPs.

The fact that some of the novel variants occur in the Kalmar population at frequencies similar to those of the candidate SNPs suggests that the novel variants and the candidate SNPs may be in linkage. In other words, it may be that individuals which, in the HRM analyses used for the present study, register as having a “southern” genotype, actually carry a Kalmar‐specific haplotype that is functionally distinct from the southern‐Swedish haplotype. Unfortunately, the pooled‐sequence dataset used here cannot be used to discern linkage farther than the length of a single read (in this case, 150 bp). Reconstructing whole‐gene alleles (haplotypes) would require individual‐level sequence data, which is logistically beyond the scope of the current study.

Although our interpretation of the results remains speculative without linkage information, it is nonetheless worth noting that the presence of novel variants at life cycle‐regulating loci makes adaptive sense. The Kalmar population experiences a relatively short season, and expresses a univoltine life cycle similar to that of other mainland populations further north (Lindestad et al., 2019). Genetic variants that cause averted or postponed diapause would likely be selected against in this environment, whether they were introgressed through gene flow from southern populations at some point in the past, or left over from the initial postglacial expansion (Lindestad et al., 2022). The fact that the southern candidate SNPs have nonetheless remained in the Kalmar population long‐term may, then, be explained by the presence of additional, geographically local mutations in *tim* and/or *per* that compensate for the effect of these southern variants: a form of within‐gene adaptive epistasis (Lehner, 2011).

The genetic basis of phenotypic plasticity has been a much‐pursued and hotly debated problem in evolutionary biology for many decades (Nicoglou, 2015). Given the drastic but highly predictable environmental differences between seasons, and the concomitant drastic changes in phenotype often produced (e.g. diapause/nondiapause), seasonal plasticity in insects is an excellent arena in which to study the evolution of plasticity (Gotthard & Nylin, 1995; Moran, 1992). The genetic variation underlying seasonal plasticity is of especially great interest because it determines in part how populations respond to ongoing climate change (Williams et al., 2017), as well as to anthropogenic movement of species between geographic regions (Grevstad & Coop, 2015). The present results suggest a caveat when undertaking such research (and when using a candidate gene approach in general), namely that correlations between genetic variants and phenotypic responses may be geographically context‐dependent, and so extrapolations (e.g. from allele frequencies across a climate cline) may lead to misleading conclusions. Although the present study failed to experimentally detect an effect of the candidate SNPs in this new geographic context, the notably large amount of nonsynonymous amino acid‐level variation in the two circadian genes studied (Figure 3) is worthy of further examination.

## AUTHOR CONTRIBUTIONS

**Olle Lindestad:** Conceptualization (equal); formal analysis (lead); investigation (lead); methodology (equal); visualization (lead); writing – original draft (lead). **Sören Nylin:** Funding acquisition (equal); supervision (supporting); writing – review and editing (supporting). **Christopher W. Wheat:** Investigation (supporting); methodology (supporting); software (supporting); supervision (equal); writing – review and editing (supporting). **Karl Gotthard:** Conceptualization (equal); funding acquisition (equal); investigation (supporting); methodology (equal); supervision (equal); writing – review and editing (equal).

## FUNDING INFORMATION

This work was financed by the Knut and Alice Wallenberg Foundation (KAW 2012.0058), the Bolin Centre for Climate Research at Stockholm University (to K. Gotthard), and the Swedish Research Council grants VR‐2012‐3715 (to S. Nylin) and VR‐2017‐04500 (to K. Gotthard).

## CONFLICT OF INTEREST STATEMENT

The authors have no conflicts of interest to declare.

## Data Availability

Experiment data and analysis scripts for this manuscript have been made available at Zenodo: https://doi.org/10.5281/zenodo.11105039.
